# Priors and proprioceptive predictions

**DOI:** 10.1016/j.cobeha.2025.101509

**Published:** 2025-06-01

**Authors:** Thomas Parr, Maxwell JD Ramstead, Karl Friston

**Affiliations:** 1 Nuffield Department of Clinical Neurosciences, University of Oxford, UK; 2 Queen Square Institute of Neurology, University College London, UK

## Abstract

This review presents an approach to motor control inspired by the Equilibrium Point Hypothesis. The core idea is that, to realise a motor plan, one need only anticipate the proprioceptive consequences of that plan. Movement can then be executed through spinal and brainstem reflex arcs that correct for any deviations from these proprioceptive predictions. Seen in this light, motor commands are proprioceptive predictions. From a control-theoretic perspective, this implies that reflexes can be cast as closed feedback loops, the set points of which are determined by proprioceptive predictions. In what follows, we consider the key elements — in terms of active inference — that generate proprioceptive predictions. These include prior beliefs about motor trajectories, their temporal (autocorrelation) structure, and the confidence with which their sensory consequences can be predicted. For each element, we briefly review the neurobiology of the structures that might support the underlying computations. In short, we will see how corticospinal, cerebellar, and extrapyramidal systems might contribute to the prediction and realisation of a motor plan.

## Introduction

Control theoretic perspectives on the neurobiology of the motor system rely upon the notion of closed-loop feedback [1]. The idea is that a set point may be specified and achieved via negative feedback mechanisms that resolve discrepancies between the state of a motor plant and the desired set point. In this review, we consider an approach inspired by the Equilibrium Point Hypothesis — which proposes that movements are generated based on motor neuron recruitment when a (partly) centrally determined threshold for a muscle length is exceeded [2]. An interpretation of this hypothesis is that spinal and brainstem reflexes play the role of closed-loop controllers, where the feedback is the proprioceptive signal communicated to the central nervous system via type Ia and II sensory afferent neurons (where the full loop includes both the reflex arc and the system being controlled). We focus on a framing of equilibrium points as *predictions about proprioceptive signals*. The equivalence between the preferred state (set point) and the predicted state is at the heart of the active inference accounts of motor behaviour [3–6]. To avoid confusion, the word *prediction* here is used in the sense of predictive processing [7]. It means the prediction we would make given a generative world model. This means it is not (necessarily) a *future* prediction but is ongoing and simultaneous with the sensory data our brain’s model seeks to explain. As predictions are refined, or as we act to change sensory data, prediction errors are resolved to bring predictions in line with current afferent input. In common with approaches more directly implementing the ideas beneath Equilibrium Point control [8], active inference approaches have shown promise in robotic systems [9–12].

The link between prediction and action brings in elements of ideomotor theory [13], which deals with the translation of ideas into motor behaviour. Ideomotor phenomena are sometimes exploited by illusionists, and the theory is often used to explain tricks such as table turning or Ouija boards of the sort used by 19th-century mediums [14–16]. Explanations are based on the idea that sufficiently strong expectations — in Bayesian terms, probabilistic prior beliefs — that movement will occur are sufficient to generate those same movements through the reflexive fulfilment of predictions about their sensory consequences.

Perceptual control theory (PCT) is a theory of the behaviour of living organisms based on negative feedback control [17,18]. The active inference approach to motor control might be seen as largely compatible with PCT [19]. However, we note other articles in this special issue argue against this notion [20]. According to PCT, motor behaviour is the control of sensory input through counteracting disturbances to internal set points (reference values). Active inference can be seen as a version of this idea but with a complementary conceptual emphasis. While PCT treats ‘perceptual signals’ as transformations of sensory data to be controlled, active inference sees percepts as Bayesian beliefs that synthesise information from priors and sensations. Belief updating (perceptual inference) and motor control are then treated as the joint optimisation of the same objective function that measures the fit between an internal model and the sensations it seeks to explain. The coupling of action and perception implicit here is shared with the Equilibrium Point and Perceptual Control approaches and helps underline the consistency of these theoretical approaches.

Another closely related scheme is ‘optimal control theory.’ The main difference between active inference and optimal control theory is that the latter calls upon both a forward (predictive) model and an inverse model that deals with feedback from the motor system and identifies the motor ‘commands’ necessary to fulfil some ‘goal’ [21]. In contrast, active inference employs only a single forward model [22], c.f., model predictive control [23], and is therefore not an example of an internal model theory of the ‘optimal control’ sort but is more in keeping with the broader interpretation of internal — a.k.a. generative or world — models implicit in Bayesian brain and predictive processing accounts in neuroscience [24,25]. The role played by an inverse model is subsumed by low-level reflex arcs in the sense that the ‘goal’ is the anticipated proprioceptive feedback and can be fulfilled through relatively simple negative feedback loops. However, one of the reasons sometimes offered for the importance of a separate inverse model is the need to account for sensorimotor delays sometimes thought to be too long to support feedback control of the equilibrium point sort [26]. The reconciliation is an important update to the Equilibrium Point Hypothesis, widely used in active inference, known as generalised coordinates of motion.

The idea behind the use of generalised coordinates of motion is that our nervous system makes use of representations not just of the current state of the motor system but of the *rates of change* of these states, that is, the velocities, accelerations, and higher temporal derivatives of relevant variables [27]. Taken together, such coordinates play a similar role to the coefficients of Taylor series expansions of trajectories. This means they can be used to project short distances into the future (c.f. ‘strong anticipation’ [28]) or past to account for delays [29]. A further advantage of this form of representation is that it provides an opportunity to account for correlations between different orders of motion and, therefore, to make sense of sensorimotor fluctuations with a nontrivial autocorrelation structure. This feature will become relevant below when we discuss a possible role for descending pathways from the cerebellar nuclei to the brainstem and spinal targets.

The theoretical frameworks outlined above have all had success in accounting for aspects of motor behaviour, and our view is that they are largely compatible with one another — albeit using different lexica. Most of these approaches have common ground in that they put a great deal of emphasis on the role of spinal and brainstem reflexes, their interaction with the peripheral nervous system, and appeal to a form of closed-loop control reliant on resolving errors or discrepancies from thresholds or setpoints. Our agenda here is to understand the neurobiological origins of the set points or — through the lens of predictive processing — proprioceptive predictions. We will pay particular attention to the role of inference about sequences that determine the trajectories of predictions (or set points) in generating complex behaviours.

In what follows, we will focus on the proprioceptive predictions that act as equilibrium points for reflex loops. First, we highlight that predictions have two important components. These are the first and second-order statistics — that is, the mode and covariance — of anticipated sensory signals. The predicted covariance is particularly important in setting the gain of reflexive control. It depends upon both the variance of sensory fluctuations and upon their autocorrelation or smoothness. Second, we focus on the content of these predictions. We consider the way in which alternative motor plans might translate into predicted proprioception and how this might be sequenced into a series of equilibrium points [30]. We unpack each of the elements — or priors contributing to these predictions in terms of the underlying computational neuroanatomy.

## Reflex arcs

**Figure 1:**
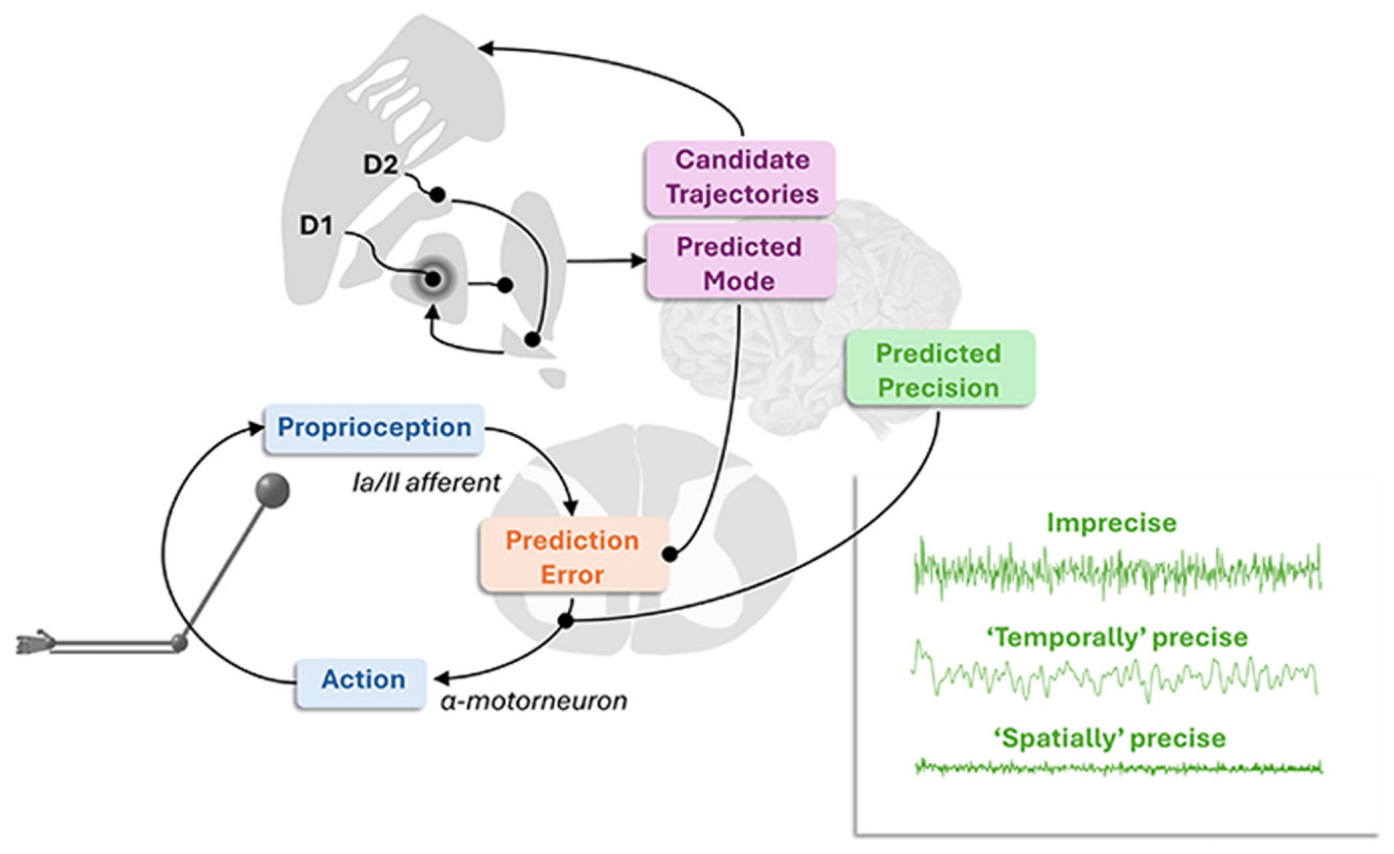
This schematic sets out, in simplified form, some of the key structures and computations outlined in this paper. Central to this is the closed-loop (reflexive) feedback system (shown in the lower left), in which sensory feedback, in the form of proprioception, is compared to the mode of descending predictions to determine the size of the error to be corrected by movement. This prediction error is contextualised by the confidence or precision of the prediction. The predicted precision involves estimates of both (‘spatial’) and autocorrelative (‘temporal’) components. The inset in the lower right of the graphic shows the difference between these two precision components by taking an imprecise noise process and increasing either the temporal or spatial components of the precision. The upper parts of the graphic set out some of the computations that may determine priors for predictions, illustrating the idea that alternative sequences of movements might be evaluated by the basal ganglia network to help determine the predictive mode communicated by the motor cortex to the spinal cord. The direct and indirect pathways, starting with the D1 and D2 receptor-expressing medium spiny neurons of the striatum, are shown as a coronal cross-section of the nuclei of the basal ganglia and thalamus. The pointed and round arrowheads indicate excitatory (glutamatergic) and inhibitory (GABAergic) synapses, respectively. Note that the D1 pathway leads to net excitation and D2 to net inhibition, but with different spatial distributions, leading to the centre-surround [31] like pattern shown in the internal globus pallidus. Balancing the two pathways sets a prior belief about the confidence in the selection of one of several alternative action plans.

**Figure 2:**
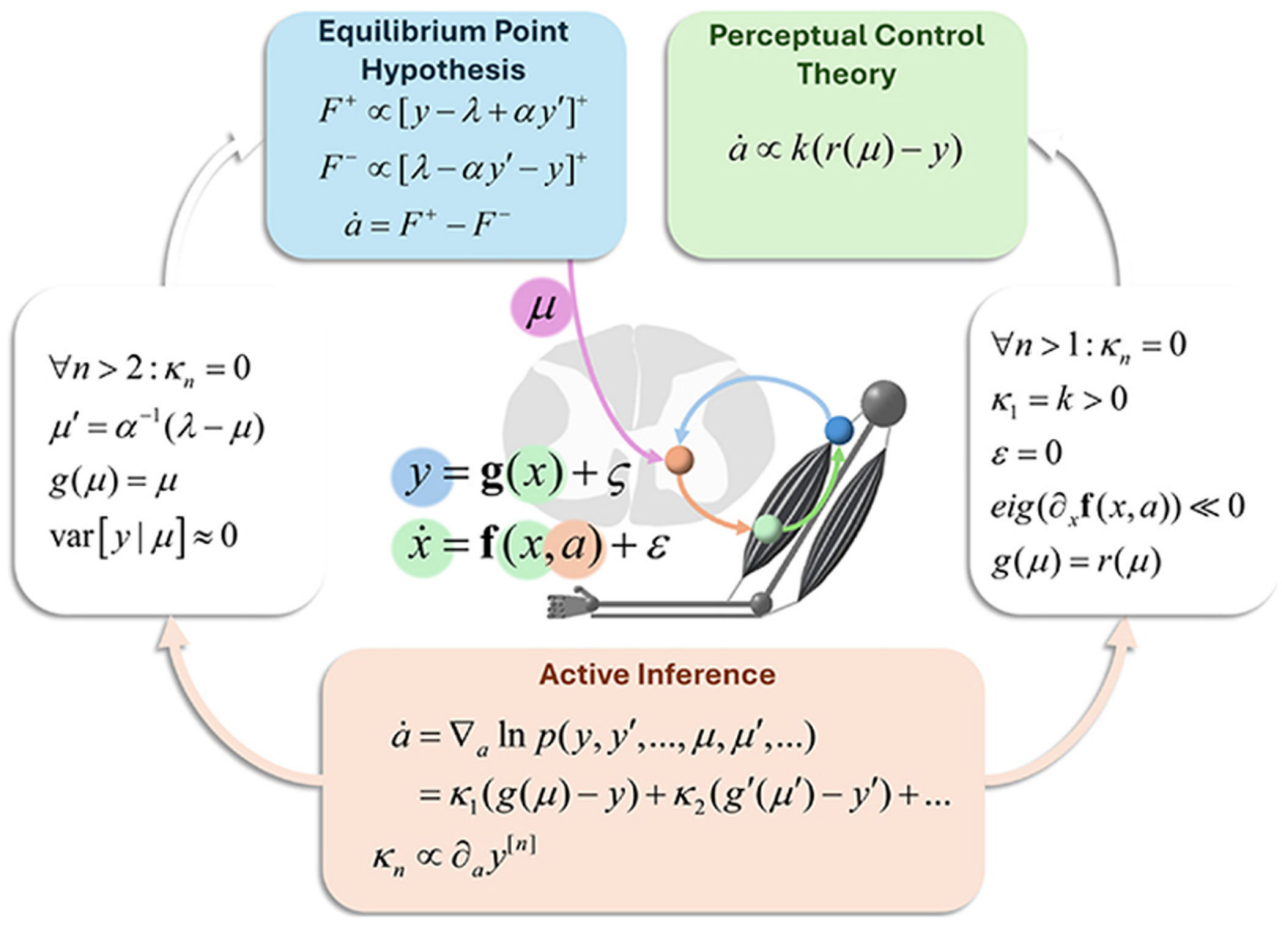
This figure supplements the conceptual account in the text with additional mathematical detail to unpack the formal relationships between the related accounts on offer — acknowledging that all three accounts here are incomplete. The central graphic sets out the key variables we are interested in and their role in a reflex arc. The variable *x* is the set of variables representing the physical state of the motor plant (e.g. muscle lengths or joint angles). The *y* variable represents the afferent sensory input (e.g. from tendon stretch receptors). The *a* represents the efferent limb of the reflex arc (i.e. motor-neuron recruitment). Finally, *μ* represents the central input to the system. The bold functions **f** and **g** give the dynamics of the motor plant and the generation of afferent signals, respectively. These are supplemented with stochastic terms (*ε* and *ς*) to reflect random contributions to the dynamics. The prime notation is used to indicate the order of generalised motion (position, velocity, etc.). The superscripted [*n*] indicates the *n-*th order of motion. The Active Inference panel shows the action as changing the afferent input such that it maximises the (log) evidence for an internal model (under a Laplace assumption [34]). The second line unpacks this so that we can interpret the ensuing dynamics as a minimisation of prediction errors (with predictions given by the function *g*). This scheme is generic to a range of control problems. By making specific assumptions about the terms in these equations, one can recover the Equilibrium Point hypothesis (as expressed in Equations 2–3 of [35], in which we have assumed the force is the net force of both agonist and antagonist and in which […]^+^ indicates the operation max(…, 0)) and the basic unit of Perceptual Control Theory (see Equations 1–3 of Ref. [36]). The key assumptions that take us to the Equilibrium Point hypothesis are that forces are generated in proportion to the positive deviation from some threshold muscle length. When this threshold is met, the resultant force is zero. This is achieved by the Active Inference scheme when the predicted velocity *μ*′ has attractor-like dynamics and when *μ* can be determined precisely from *y*. The Perceptual Control scheme emerges when the plant dynamics relax very quickly (i.e. the eigenvalues of the Jacobian are very negative) to an attracting state determined by *a* (in other words, *y* is effectively a direct function of *a*). This ensures that only the first term in the sum in the Active Inference panel is needed, giving a simple form for set-point control.

Figure 1 illustrates the basic structure of a reflex arc and, implicitly, the control problem that we are interested in. Figure 2 unpacks this in greater mathematical detail. It depicts sensory afferent fibres of the II and Ia types carrying proprioceptive (i.e. length and rate of change of length of muscle tendons) signals from muscle to the dorsal root of the spinal cord. This incoming signal is compared with predictions from the descending motor tracts. Physiologically, ‘comparison’ here means the subtraction of inhibitory (e.g. GABAergic) from excitatory (e.g. glutamatergic) inputs to the post-synaptic — membrane yielding no change in post-synaptic membrane potential when the descending and afferent signals match. The discrepancy between the prediction and the sensory signal determines the activity of an α-motor neuron in the ventral horn of the spinal cord. The axon of this motor neuron terminates in the muscle, causing changes in muscle contraction when the proprioceptive signal does not match the prediction. This means muscle contraction depends upon both the proprioceptive input *and* the predicted input. At the point that the prediction is fulfilled — that is, when the afferent signal is brought in line with the prediction — the resulting net force is zero. The descending prediction includes predictions (or thresholds — see Refs. [32,33]) about the expected proprioceptive signal and the confidence of this expectation. The confidence, precision, or inverse uncertainty is a gain signal that multiplicatively weights the effect of prediction error on the motor output.

In the next section, we will consider in more detail the form of the expected proprioceptive signal itself. Here, we will focus on the precision associated with the prediction. This precision decomposes into two parts [37]. Heuristically, these can be cast as a spatial and a temporal component. The spatial component represents the inverse covariance of a signal at a given point in time. The temporal component depends upon the auto-correlation — that is, the smoothness — of the signal over time. The graphic in the lower right portion of Figure 1 illustrates this point by showing the effect of increasing either the spatial or temporal components of the precision of a noise process.

The consequences of getting these precisions wrong [38] give us clues as to their neuroanatomical substrates. Overestimation of the spatial component of the precision leads to a much faster resolution of prediction error and, so, to a brisker response to an unexpected proprioceptive signal — of the sort that might be elicited tapping on a muscle tendon. This is consistent with the clinical phenotype associated with upper motor neuron lesions. Such lesions affect the corticospinal tract and occur in a wide range of pathologies, including motor neuron disease [39], multiple sclerosis [40], and stroke [41]. The implication is that part of the role of descending corticospinal projections is to attenuate the precision or gain of low-level reflex arcs [42]. Interestingly, Equilibrium Point theorists have articulated a subtly different account of post-stroke spasticity, in which it is the threshold parameter itself that is reduced such that the equilibrium (or referent) muscle lengths are shorter than they would otherwise be [43].

Hyperreflexia of this sort is not limited to discrete structural lesions. Interestingly, an autoimmune disorder known as stiff person syndrome [44], in which auto-antibodies are directed against glutamate decarboxylase (GAD) in the dorsal horn of the spinal cord, also presents with exaggerated reflexes. One of the roles of GAD is to synthesise the inhibitory neurotransmitter GABA. The idea that some descending corticospinal projections attenuate precision or gain is reinforced by the observation that autoimmune destruction of inhibitory synapses, at the target site of these projections, results in the same excessive motor response to sensory feedback.

The effect of overestimation of the temporal component of precision is slightly subtler. This deals with correlations between different orders of motion — for example, between the current position and acceleration of some variable [45]. A negative correlation between position and acceleration in a dynamical system implies pendular or oscillatory dynamics. Simulation studies [38] confirm that excessive smoothness estimates lead to oscillatory reflex patterns and slow periodic fluctuations imposed upon reaching movements. These closely resemble those seen in cerebellar syndromes [46] and implicate descending cerebellospinal tracts (i.e. those corticospinal, vestibulospinal, and rubrospinal axons influenced by the activity of deep cerebellar nuclei) in the modulation of estimates of temporal precision.

A final note on this control loop concerns what happens when the feedback from proprioception is impaired. An example of this is in sensory neuronopathies — characteristically associated with Sjogren’s syndrome [47] — in which the cell bodies of proprioceptive neurons become inflamed. Characteristically, this causes a pseudoathetosis, in which smooth tremulous movements in outstretched limbs can emerge on eye closure [48]. The implication of this is that predictions and errors from other sensory modalities (e.g. vision) can compensate for the loss of precise proprioceptive data. However, when sensory feedback is inadequate, the smooth fluctuations implicit in proprioceptive predictions are unchecked and — given the equivalence between predictions and commands — communicated to muscles. This might manifest in the enaction of these predictions and the enaction of these smooth tremulous movements.

## Descending predictions

Our previous section dealt with the issue of predicting the appropriate precision of fluctuations in proprioceptive predictions. We now turn to the question of how those predictions might be determined by supratentorial structures in the brain. For a related account of the underlying dynamics generating trajectories of setpoints, involving transitions between resting attracting states and dynamic targets (see Ref. [49]). This leads to the consideration of both cortical and subcortical structures. Starting with the former, the site of origin of descending corticospinal projections is (mostly) Layer V (Betz) pyramidal cells of the primary motor cortex [50], Brodmann area 4. The motor cortex has several interesting cytoarchitectural features of relevance for predictive motor control. The first is the relative lack of cells in Layer IV [51]. This cortical layer, in many other cortical areas, is populated by granular spiny stellate cells in receipt of ascending projections from primary thalamic nuclei and cortical regions closer to primary sensory cortices [52]. For this reason, it is often thought that projections to Layer IV communicate prediction errors up the cortical hierarchy. The relative paucity of cells in this layer in the primary motor cortex endorses the idea that errors in predictions made by the motor cortex are typically resolved at the spinal cord or brainstem level — that is, through reflexive feedback control — such that there is minimal residual prediction error to pass back up to the cortex [3].

We have discussed the confidence in motor cortical predictions, but what about their content? There is an emerging view, rooted in the concept of motor chunking [53], that these predictions should anticipate sequences of short motor trajectories that can be combined to produce complex movements [54]. There is compelling electrophysiological evidence for this, including the demonstration that populations of neurons in the motor cortex fire maximally for specific reaching trajectories [55]. Furthermore, the supplementary motor cortex contains populations whose activity — projected onto a low dimensional space — follows periodic trajectories with variable frequencies [56], some of which can be entrained by periodic sensory stimuli. These might act as internal clocks to help sequence a set of equilibrium points.

While the motor cortex and its neighbours may be capable of representing alternative trajectories and the ways in which these might be sequenced, it is important to be able to select among the alternatives. Another pair of cytoarchitectural features hints at the networks involved in this process. These are the projections from Layer V Betz cells to the basal ganglia (specifically, to the striatum and subthalamic nucleus) and the modulation of dendrites in superficial cortical layers by basal ganglia output nuclei via the thalamus [57]. The implication is that cortico-subcortical loops involving the basal ganglia may be involved in the selection and timing of sequences of motor trajectories.

Pathology again gives us clues as to the role of sub-cortical structures. For instance, Parkinson’s disease involves the degeneration of dopaminergic projections from the midbrain to the striatum, which act to balance the relative contribution (via direct and indirect pathways) of D1 and D2 receptor-expressing medium spiny neurons (MSNs) to the basal ganglia outputs. D1-MSNs have large dendritic trees and evoke spatially precise activity changes in the output nuclei, while D2-MSNs have smaller dendritic trees and evoke broader patterns of change in output nuclei [58]. Assuming the basal ganglia outputs represent alternative action plans, the balance between these two MSN populations determines how confident we can be in selecting a specific plan, with D1-MSNs using their extensive dendritic inputs [59] to provide precise beliefs about the best policy, countering the coarser suppression of alternative policies by D2-MSNs. Loss of confidence might underwrite the paucity of movement in Parkinson’s disease. In contrast, other pathologies can cause hyperkinetic movements, such as the dyskinesias of both Huntington’s disease and those that sometimes result from dopaminergic therapies, which present as involuntary but fluent dance-like movements [60]. This is consistent with the idea that disruptions of the striatal MSN pathways can also lead to overconfidence in the selection of unintended action sequences that are nonetheless internally consistent sequences. Please see Ref. [61] for a discussion of electrophysiological beta activity and its putative encoding of precision.

In addition to affecting confidence in which movement to select, Parkinsonism can present with arrhythmokinesis [62], in which patients struggle to maintain consistent beat-to-beat intervals in repetitive movements — such as finger tapping. Various forms of sensory cueing — typically those with a degree of spatial or temporal regularity [63], such as a metronome beat or stripes painted on the floor [64] — can often be used to restore confidence in timing and to lead to more fluent movement control: see Refs. [65,66] for related computational studies. Plausibly, the importance of temporal precision offers a role for the hyperdirect basal ganglia pathway that bypasses the striatum and targets the subthalamic nucleus — interestingly also a target for deep brain stimulation to relieve symptoms of Parkinson’s disease [67]. Taken together, pathological observations in disorders of the basal ganglia hint at a role in selecting between alternative sequences and timings of steps of equilibrium points that lead to the proprioceptive predictions the motor cortex communicates to spinal reflex arcs.

## Conclusion

The above overview of control theoretic aspects of motor control has focused on the supratentorial structures that might determine proprioceptive predictions that act as set points for closed-loop feedback control enacted by infratentorial structures. This appeals to a version of the Equilibrium Point Hypothesis [68], in which sequences of movement chunks are selected and enacted by actively resolving errors between proprioceptive predictions and incoming data to the spinal cord. This notion makes sense of several cytoarchitectural features of the networks for motor control and has important implications for how we might understand the computational underpinnings of disorders of motor control.

## References

[1] Marken RS, Mansell W (2013). Perceptual control as a unifying concept in psychology. Rev Gen Psychol.

[2] Feldman AG, Levin MF, Sternad D (2009). Progress in Motor Control: A Multidisciplinary Perspective.

[3] Adams RA, Shipp S, Friston KJ (2013). Predictions not commands: active inference in the motor system. Brain Struct Funct.

[4] Hipólito I (2021). Embodied skillful performance: where the action is. Synthese.

[5] Friston KJ (2010). Action and behavior: a free-energy formulation. Biol Cyber.

[6] Friston K, Mattout J, Kilner J (2011). Action understanding and active inference. Biol Cybern.

[7] Rao RP, Ballard DH (1999). Predictive coding in the visual cortex: a functional interpretation of some extra-classical receptive-field effects. Nat Neurosci.

[8] Pagnanelli G (2024). Integrating human-like impedance regulation and model-based approaches for compliance discrimination via biomimetic optical tactile sensors. IEEE Trans Robot.

[9] Tani J, White J (2020). Cognitive neurorobotics and self in the shared world, a focused review of ongoing research. Adapt Behav.

[10] Pio-Lopez L (2016). Active inference and robot control: a case study. J R Soc Interface.

[11] Bos F (2022). Free energy principle for state and input estimation of a quadcopter flying in wind.

[12] Çatal O (2021). Robot navigation as hierarchical active inference. Neural Netw.

[13] Shin YK, Proctor RW, Capaldi EJ (2010). A review of contemporary ideomotor theory. Psychol Bull.

[14] Andersen M (2019). Predictive minds in Ouija board sessions. Phenomenol Cogn Sci.

[15] Carpenter WB (1852). On the influence of suggestion in modifying and directing muscular movement, independently of volition.

[16] Faraday M (1838). Experimental investigation of table-moving. J Franklin Inst.

[17] Powers WT (1973). Behavior: The Control of Perception.

[18] Mansell W (2020). The Interdisciplinary Handbook of Perceptual Control Theory: Living Control Systems IV.

[19] Pezzulo G, Parr T, Friston K (2021). The evolution of brain architectures for predictive coding and active inference. Philos Trans R Soc B Biol Sci.

[20] Mansell W, Gulrez T, Landman M (2025). The prediction illusion: perceptual control mechanisms that fool the observer. Curr Opin Behav Sci.

[21] Wolpert DM, Kawato M (1998). Multiple paired forward and inverse models for motor control. Neural Netw.

[22] Friston K (2011). What is optimal about motor control?. Neuron.

[23] Schwenzer M (2021). Review on model predictive control: an engineering perspective. Int J Adv Manuf Technol.

[24] Knill DC, Pouget A (2004). The Bayesian brain: the role of uncertainty in neural coding and computation. Trends Neurosci.

[25] Doya K (2007). Bayesian Brain: Probabilistic Approaches to Neural Coding.

[26] Kawato M, Wolpert D, Bock GR, Goode JA (2007). Internal models for motor control.

[27] Friston K (2008). Hierarchical models in the brain. PLoS Comput Biol.

[28] Stepp N, Turvey MT (2010). On strong anticipation. Cogn Syst Res.

[29] Perrinet LU, Adams RA, Friston KJ (2014). Active inference, eye movements and oculomotor delays. Biol Cybern.

[30] Rabinovich M, Bick C, Varona P (2023). Beyond neurons and spikes: cognon, the hierarchical dynamical unit of thought. Cogn Neurodyn.

[31] Nambu A (2004). Progress in Brain Research.

[32] Raptis H (2010). Control of wrist position and muscle relaxation by shifting spatial frames of reference for motoneuronal recruitment: possible involvement of corticospinal pathways. J Physiol.

[33] Feldman AG, Orlovsky GN (1972). The influence of different descending systems on the tonic stretch reflex in the cat. Exp Neurol.

[34] Zeidman P, Friston K, Parr T (2023). A primer on Variational Laplace (VL). NeuroImage.

[35] Feldman AG, Levin MF (2024). Progress in Motor Control.

[36] Powers WT, Clark RK, McFarland RL (1960). A general feedback theory of human behavior. Part I. Percept Mot Skills.

[37] Friston K (2010). Generalised filtering. Math Probl Eng.

[38] Parr T (2021). The computational neurology of movement under active inference. Brain.

[39] Swash M (2020). Occasional essay: upper motor neuron syndrome in amyotrophic lateral sclerosis. J Neurol Neurosurg Psychiatry.

[40] Ford H (2020). Clinical presentation and diagnosis of multiple sclerosis. Clin Med.

[41] Wilson LR (1999). Muscle spindle activity in the affected upper limb after a unilateral stroke. Brain.

[42] Brown H (2013). Active inference, sensory attenuation and illusions. Cogn Process.

[43] Piscitelli D (2020). Deficits in corticospinal control of stretch reflex thresholds in stroke: implications for motor impairment. Clin Neurophysiol.

[44] Rakocevic G, Floeter MK (2012). Autoimmune stiff person syndrome and related myelopathies: understanding of electrophysiological and immunological processes. Muscle Nerve.

[45] Bhashyam B, Friston K (2011). Bayesian state estimation using generalized coordinates. Proc Spie.

[46] Holmes G (1917). The symptoms of acute cerebellar injuries due to gunshot injuries. Brain.

[47] Mori K (2005). The wide spectrum of clinical manifestations in Sjögren’s syndrome-associated neuropathy. Brain.

[48] Lo YL, See S (2010). Images in clinical medicine. Pseudoathetosis. N Engl J Med.

[49] Martin V, Reimann H, Schöner G (2019). A process account of the uncontrolled manifold structure of joint space variance in pointing movements. Biol Cybern.

[50] Nolan M (2024). Betz cells of the primary motor cortex. J Comp Neurol.

[51] Shipp S, Adams RA, Friston KJ (2013). Reflections on agranular architecture: predictive coding in the motor cortex. Trends Neurosci.

[52] Zeki S, Shipp S (1988). The functional logic of cortical connections. Nature.

[53] Wymbs NF (2012). Differential recruitment of the sensorimotor putamen and frontoparietal cortex during motor chunking in humans. Neuron.

[54] Friston KJ, Parr T, de Vries B (2017). The graphical brain: belief propagation and active inference. Netw Neurosci.

[55] Georgopoulos AP, Schwartz AB, Kettner RE (1986). Neuronal population coding of movement direction. Science.

[56] Gámez J (2019). The amplitude in periodic neural state trajectories underlies the tempo of rhythmic tapping. PLoS Biol.

[57] Shipp S (2007). Structure and function of the cerebral cortex. Curr Biol.

[58] Gertler TS, Chan CS, Surmeier DJ (2008). Dichotomous anatomical properties of adult striatal medium spiny neurons. J Neurosci.

[59] Wall NR (2013). Differential innervation of direct- and indirect-pathway striatal projection neurons. Neuron.

[60] Vitale C (2001). Unawareness of dyskinesias in Parkinson’s and Huntington’s diseases. Neurol Sci.

[61] Palmer CE (2019). Sensorimotor beta power reflects the precision-weighting afforded to sensory prediction errors. Neuroimage.

[62] Trager MH (2015). Arrhythmokinesis is evident during unimanual not bimanual finger tapping in Parkinson’s disease. J Clin Mov Disord.

[63] Rahman S (2008). The factors that induce or overcome freezing of gait in Parkinson’s disease. Behav Neurol.

[64] Janssen S (2016). A painted staircase illusion to alleviate freezing of gait in Parkinson’s disease. J Neurol.

[65] Friston KJ (2012). Dopamine, affordance and active inference. PLoS Comput Biol.

[66] Parr T, Oswal A, Manohar SG (2025). Inferring when to move. Neurosci Biobehav Rev.

[67] Gulberti A (2024). Premotor cortical beta synchronization and the network neuromodulation of externally paced finger tapping in Parkinson’s disease. Neurobiol Dis.

[68] Feldman AG (2009). New insights into action-perception coupling. Exp Brain Res.

